# Gel nail polish as an alternative to traditional coverslip sealants: A quick solution to a sticky situation

**DOI:** 10.1016/j.mex.2023.102256

**Published:** 2023-06-14

**Authors:** Gabriela Kennedy, Daryan Chitsaz, Jeanne F. Madranges, Melissa Peştemalciyan, Abbas F. Sadikot, Timothy E. Kennedy

**Affiliations:** Department of Neurology & Neurosurgery, Montreal Neurological Institute, 3801 Rue University, McGill University, Montréal, QC, Canada H3A 2B4

**Keywords:** Histology, Histological analysis, Cover glass, Mounting medium, Microscope slide, Rapid Coverslip Sealing

## Abstract

A widespread protocol to seal coverslips on a microscope slide for histological analysis utilizes air-drying nail polish. Nail polish is applied to glue the coverslip in place and prevent the leakage of mounting media. Air drying takes time, typically overnight, and generates an unpleasant smell. Equally familiar is the waiting game, lightly touching the polish to check its dryness, while being careful not to disrupt the coverslip, often leaving sticky spots on one's fingertips. An advantageous solution to these drawbacks is to use gel nail polish, which rapidly hardens and dries by being cured under a LED/UV lamp. We show that UV-cured gel nail polish provides a rapid, stable, scentless, nontoxic, and cost-effective solution for coverslip sealing. Cured in 10 s, with no impact on fluorescent labels, gel polish hardens completely and the slide is ready to be imaged. Furthermore, we show that gel nail polish can be used to generate 3D ridges and structures to support coverslipping thicker samples. Gel nail polish is purposefully unscented, and the brands used in our study employ environmentally conscious, vegan, and cruelty-free ingredients. UV-cured gel nail polish is a cost-effective alternative that presents an easy, accessible, and inexpensive solution to traditional coverslip sealing methods.

• Inexpensive method to rapidly seal coverslips onto a microscope slide to immediately image samples for Histological analyses.
• Utilizes LED/UV light to cure gel nail polish in 10 s without bleaching fluorophores.
• Can be used to generate 3D ridges and structures to support coverslipping thicker samples.

## Graphical abstract

**Figure:**
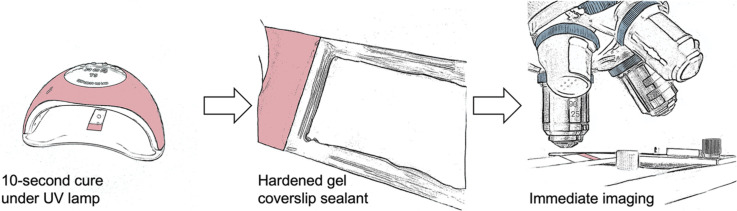

Specifications table

**Table:** 

Subject area:	Agricultural and Biological Sciences
More specific subject area:	Histology and Microscopy
Name of your method:	Rapid Coverslip Sealing
Name and reference of original method:	Donaldson, J.G. (2015). Immunofluorescence Staining. Curr Protoc Cell Biol *69*, 431–437.
Resource availability:	Gel-curing LED/UV lamp; Gel nail polish; microscope slides and coverslips.

## Introduction

Historically, a widespread standard protocol to seal coverslips on a microscope slide for histological analysis has utilized air-drying nail polish. In this method, nail polish is applied to glue the coverslip in place and prevent the leakage of mounting media [1]. Following its application, the sealed slide is then left to air-dry prior to imaging. However, many issues and annoyances regularly arise when using air-drying nail polish as a slide sealant. Drying takes time, typically overnight, and generates an unpleasant smell. The use of air-drying nail polish can also quickly become a messy and rather sticky endeavor especially when it comes to checking whether the slide has yet dried. The relatively slow drying can be a particular problem for non-hardening mounting media that may leak while the polish sets, resulting in tilted coverslips. Furthermore, the acetone in commercial air-dry nail polish can diffuse into aqueous mounting medium and damage the sample or fluorescent dyes.

These issues have been present for as long as air-drying nail polish has been applied and are not unique to the laboratory environment. Commercial nail technicians worldwide switched to gel nail polish decades ago in the 1980′s to avoid the aforementioned complications. Gel nail polish is rapidly cured under a LED/UV lamp which hardens and dries the gel in under 10 s, effectively addressing many of the qualms experienced by air-dry nail polish users.

Gel nail polish presents a rapid, scentless, nontoxic, and cost-effective solution to coverslip sealing. Cured in 10 s, with no impact on fluorescent labels, gel polish hardens completely, and the slide can be imaged immediately. Furthermore, we demonstrate that gel nail polish can be used to rapidly generate 3D ridges and structures to support coverslipping thicker tissue sections and samples. Gel nail polish is purposefully unscented, and the brand used in our study is composed of environmentally conscious, vegan, and cruelty-free ingredients. UV-cured gel nail polish is a cost-effective alternative that presents an easy, accessible, and affordable solution to traditional coverslip sealing methods.

## Method details

### Immunohistochemistry

All procedures with animals were performed in accordance with the Canadian Council on Animal Care guidelines for the use of animals in research. Chicken embryos were incubated at 39 °C until harvesting. Stage 23 chick embryos [3] were fixed by immersion in Carnoy's solution and then embedded in paraffin blocks. Paraffin sections, 12 µm thick, were cut using a microtome onto Superfrost Plus slides (Fisher Scientific, Montreal), dewaxed at 60 °C in an oven, then rehydrated sequentially in xylene, 100% EtOH, 95% EtOH, 70% EtOH, and PBS. Tissue sections were then washed in PBS and blocked in PBS with 5% heat-inactivated horse serum and 0.1% Triton-100X prior to overnight labeling at 4 °C with a mouse anti-Beta tubulin III antibody (TUJ1, 1:1000, BioLegend, San Diego). After washes, the sections were labeled with an anti-mouse Alexa-488 conjugated secondary antibody (1:1000 dilution, Jackson ImmunoResearch Labs, West Grove, PA) and Hoechst 33,342 dye (ThermoFisher) for 1 hour at room temperature (22 °C).

### Coverslip sealing

Glass microscope slides were coverslipped using 24×50 mm cover glasses and mounting medium (Fluoromount-G, Invitrogen, Burlington). All excess mounting medium was wiped off the edges of the slides by gently tilting them onto a Kimwipe tissue. The slides were then each sealed using one of three sealing conditions. For each condition, a plastic 200 µl pipette tip with approximately 5 mm cut off its tip was used to pipette the polish onto the edges of the coverslip. All conditions employed the use of clear finishing polishes called *top coats*. The first condition employed the use of a traditional air-drying top coat (Sally Hansen Complete Salon Manicure Fast Dry Top Coat, Sally Hansen, NY). It was applied and left to dry completely in the dark. In the second and third conditions Top Gelcoat X (Aprés Nail, City of Industry, CA) or Color Gel 001 Jet Black (Leafgel, Toronto, Canada) was used as a sealant and the slides were placed in a gel-curing apparatus under UV light (SUN T5 2-in-1 75 W LED/UV Lamp, China) for 10 or 60 s, respectively. Once cured, the slides were completely dry to the touch. Slides were imaged using a Zeiss Axio Observer widefield microscope (Carl Zeiss Canada, Toronto). The intensity of the 350 nm and 488 nm excited fluorophores was measured using ImageJ [5] to visualize samples and detect bleaching.

### Parameters tested for gel nail polish hardening

**Fig. 1:**
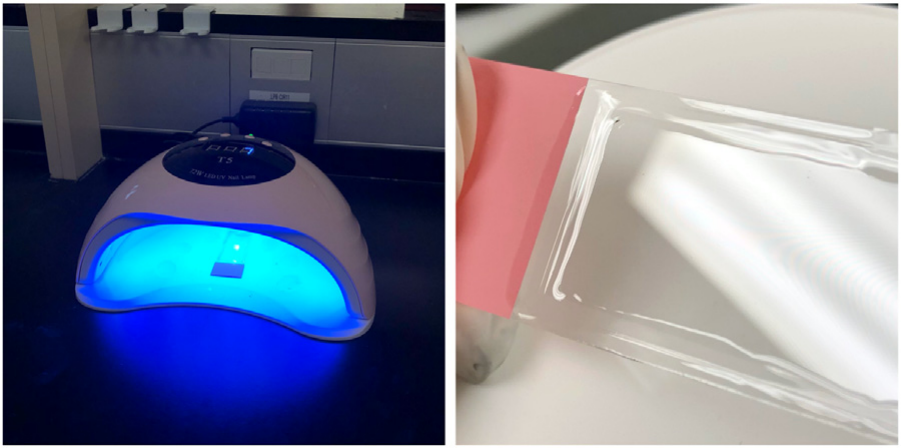
Gel-curing apparatus and gel-cured slide. Left Panel: The gel-curing apparatus (SUN T5 2-in-1 72 W LED/UV Lamp) features 3 different time settings (10, 30, and 60 s). The gel-cured top coat is completely solid to the touch following a 10 second cure. The apparatus functions by placing a coverslipped and sealed slide into the machine, selecting the curing time, and leaving the slide to cure under the light. Right Panel: Featured is a coverslipped and UV-cured gel nail polish sealed slide. This slide was cured under the LED/UV gel-curing apparatus for 10 s and was completely dry to the touch.

Gel nail polish was dried by curing in an LED/UV lamp equipped with three default curing times, 10 s, 30 s, and 60 s (Fig. 1). Some variability exists across LED/UV lamp brands in terms of default curing times, with many including the addition of a 90 second cure. For most applications, 10, 30, and 60 second curing times are standard. In all cases tested here, the relatively brief 10 second cure time was found to be effective and sufficient to completely harden the gel polish so that it was dry to the touch and ready for microscope imaging. While it is likely that many different UV light sources will work for this purpose, the type of LED/UV lamp used here is inexpensive, widely available, and small enough to be stored on a shelf or in a drawer.

**Fig. 2:**
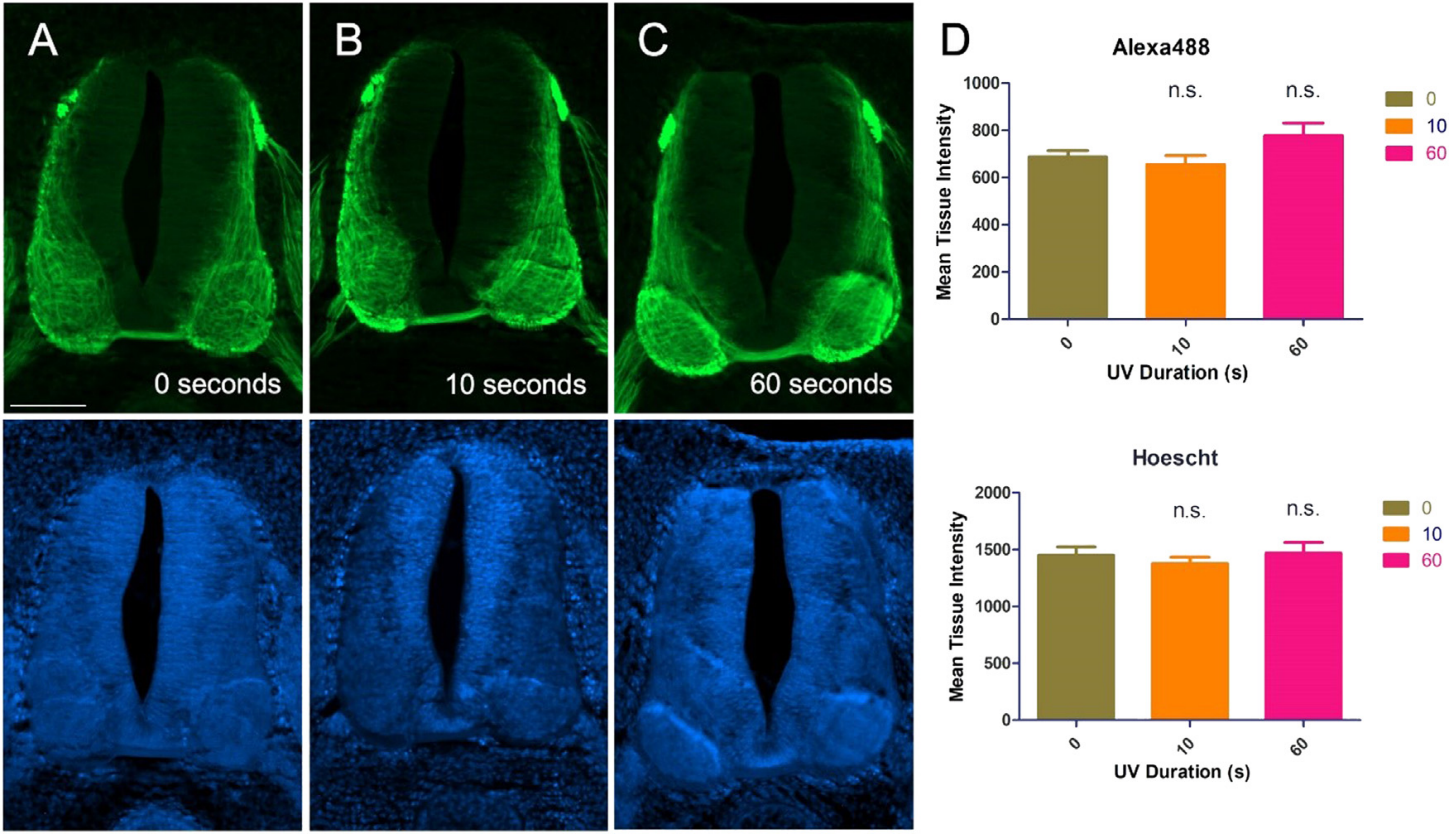
LED/UV gel lamp exposure does not diminish immunofluorescent labeling. Immunohistochemically labeled images show sections of brachial Stg23 embryonic chick spinal cord, immunolabeled with TUJ1 monoclonal antibody to mark axons (green, upper panel) and the same section counterstained with Hoechst 33,342 dye (blue, lower panel). (A) An air-drying top coat was piped onto the edges of the coverslip and air-dried overnight at room temperature. To protect against bleaching, the fluorescent immunolabeled slide was left to dry in the dark. (B, C) Coverslips were sealed onto slides using Top Gelcoat X from the brand Aprés Nail and cured using an LED/UV gel-curing lamp (T5 2-in-1 LED/UV Lamp). Slides were exposed to the LED/UV lamp for (B) 10 s and (C) 60 s. Quantification of fluorescence intensity using Fiji (Image J) indicates that exposure of the slides to the LED/UV gel-curing lamp did not significantly affect fluorescence (0 s *n* = 9 sections, 10 s *n* = 7 sections, 60 s *n* = 5 sections, one-way ANOVA, Prism 5, Scale bar in *A* = 200 µm).

Prior to testing, our main concern with this new method was the potential for the LED/UV lamp to bleach the fluorophores on the labeled slide. According to the manufacturer's description, the light emitted by the LED lamp emits wavelengths predominantly between 365 and 405 nm. To determine if bleaching was an issue, the labeled fluorescent tissue sections on slides were cured under the lamp for 10 s or 60 s. Following this LED/UV light exposure, each slide was imaged and the pixel intensity distribution across sections compared. No detectable bleaching of the Alexa-488 conjugated secondary antibodies or Hoechst 33,342 dye was detected with the use of this method (Fig. 2).

Like some air drying nail polishes, UV-cured nail polishes can exhibit autofluorescence (Express Gel: Sensational: Talk To The Palm; Sally Hansen: Salon Gel Polish: 0323 or 2224). Potential autofluorescence can be easily assessed by applying spots onto a microscope slide and imaging. When imaging a sample, nail polish should always be applied away from areas of the slide that will be imaged. If a sealed coverslip needs to be removed, the hardened gel nail polish can be solubilized with acetone, cut with a razor blade, or filed off.  Gel nail polish is composed of methyl acrylate, photo-initiators and plasticizers that provide a strong, flexible matrix when cured by UV light [4]. When used to seal coverslips, gel nail polish is long-lasting and stable. In practice, gel nail polish used to seal coverslips more than a year previously exhibited no evidence of degradation. We anticipate that this technique is readily applicable to microscopic specimens that require long-term preservation.

### Creating 3D structures

Certain gel nail polishes have been developed solely for the creation of 3D structures, and they are commonly referred to as *builder gels* or *extend gels.* Traditionally, they are used to harden and strengthen natural nails, or to sculpt false nail extensions or shapes. In this way, UV-cured gel nail polish allows for the creation of 3D structures on microscopy slides. Among many possible applications, this approach can be used to facilitate the mounting of relatively thick sections of tissue, organoids, or small whole embryos and explants.

**Fig. 3:**
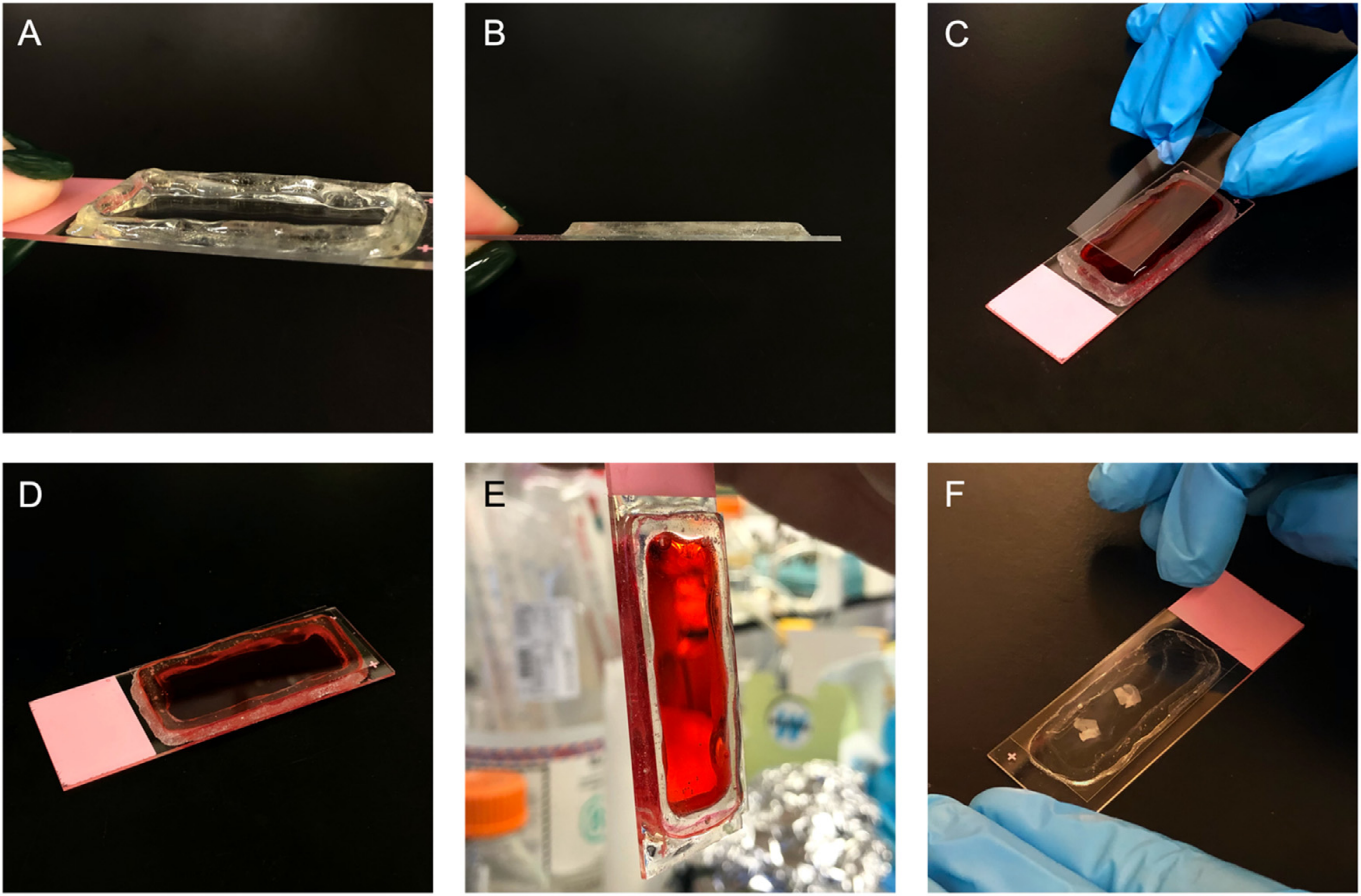
Creating a 3D structure using extend gel. (A) Layers of Extend Gel were applied to the slide with its polish brush, cured for 60 s, and wiped with 70% ethanol. This process was repeated until the desired thickness of the gel structure was achieved. (B) The surface of the gel structure was then filed flat using a 100-grit nail file. (C) Liquid extend gel was then applied to the top of the filed structure to glue down the coverslip. To test the chamber, the gel structure was filled with dyed distilled water, and then coverslipped. The structure was then cured under the LED/UV lamp for 30 s in order to adhere the coverslip to the gel structure. Following this, Extend Gel was applied to the edges of the gel structure to fill any gaps potentially remaining. It was cured once again for 30 s. (D, E) The slide was completely sealed with no leakage. (F) This process was then repeated to mount 300 µm thick sections of fixed mouse brain hippocampus.

To create a 3D structure capable of encapsulating a thick tissue sample, Extend Gel (Aprés Nail, City of Industry, CA) was applied to the edges of a coverslip and cured in the LED/UV lamp for 30 s. Once cured, the layer of gel was wiped with 70% ethanol, and a second layer was then applied. This process was repeated until the desired thickness was achieved (Fig. 3). It is important to note that Extend Gel remains sticky to the touch until the top coat is layered over it and cured. While wiping with 70% ethanol reduces the stickiness substantially, Extend Gel is never truly smooth until it is layered with a top coat (for example, Top Gelcoat X by Aprés Nail).

Following the creation of the gel wall, a nail file was used to flatten the structure. It is important to note that nail files come in different *grit grades*, referring to the coarseness of the file. The lower the number, the coarser the grit level. In this case, a 100-grit nail file was used (Nail File 100/180 Grit by Aprés Nail).

Once flat, Extend Gel was applied with its brush onto the gel structure in order to glue down the coverslip. Mounting medium (Fluoromount-G, Invitrogen, Burlington) was dropped into the well formed by the polymerized Extend Gel structure. Adult mouse hippocampal slices (300 µm-thick) were sectioned as described [2], fixed by immersion in 4% paraformaldehyde in PBS, and placed into the mounting medium. A coverslip was then placed over the applied gel on the structure and cured in the lamp for 30 s. This allowed the Extend Gel to solidify completely, adhering the coverslip to the gel structure. Following this, Extend Gel was applied to the edges of the 3D structure in the space between the coverslip and the slide in order to seal any remaining gaps (Fig. 3). The slide was then cured for an additional 30 s, which allowed it to become completely sealed and ready to image.

## Conclusions

Here we demonstrate that UV-cured gel nail polish is a rapid, scentless, nontoxic, and cost-effective solution for coverslip sealing. Using a standard labeling protocol, samples, and antibodies, we demonstrate that there is no significant bleaching of UV and blue light excited dyes by the gel-curing lamp even following 60 s of exposure. Because the gel nail polish cures nearly instantaneously, we detected little-to-no leakage under coverslips into the mounting media, as often happens with traditional sealants. This time-effective protocol produced slides that were ready to image immediately after mounting rather than needing hours to dry. We conclude that gel nail polish is a cleaner, faster, inexpensive, and easily accessible alternative to traditional sealants that can be adapted to a wide range of applications.

## Ethics statements

All procedures with animals were performed in compliance with ARRIVE guidelines in accordance with the Canadian Council on Animal Care guidelines for the use of animals in research and the National Institutes of Health guide for the care and use of laboratory animals (NIH Publications No. 8023, revised 1978).

## CRediT authorship contribution statement

**Gabriela Kennedy:** Conceptualization, Methodology, Visualization, Investigation, Writing – original draft, Writing – review & editing. **Daryan Chitsaz:** Visualization, Formal analysis, Investigation, Writing – review & editing. **Jeanne F. Madranges:** Writing – review & editing, Investigation. **Melissa Peştemalciyan:** Writing – review & editing, Investigation. **Abbas F. Sadikot:** Writing – review & editing, Supervision, Resources, Funding acquisition. **Timothy E. Kennedy:** Conceptualization, Writing – review & editing, Supervision, Resources, Funding acquisition, Project administration.

## Declaration of Competing Interest

The authors declare that they have no known competing financial interests or personal relationships that could have appeared to influence the work reported in this paper.
